# Integrating substance use training into social work education

**DOI:** 10.1186/1940-0640-10-S1-A49

**Published:** 2015-02-20

**Authors:** Marianne Pugatch, Jennifer Putney, Kimberly H McManama O’Brien, Lily Rabinow, Elissa Weitzman, Sharon Levy

**Affiliations:** 1 Adolescent Substance Abuse Program, Boston Children’s Hospital, Boston, MA, 02215, USA; 2 The Heller School for Social Policy and Management, Brandeis University, Waltham, MA, 02454, USA; 3 Simmons School of Social Work, Simmons College, Boston, MA, 02215, USA; 4 Department of Psychiatry, Harvard Medical School, Boston, MA, 02215, USA; 5 Department of Psychiatry, Boston Children’s Hospital, Boston, MA, 02215, USA; 6 Division of Adolescent/Young Adult Medicine, Boston Children’s Hospital, Boston, MA, 02215, USA; 7 Department of Pediatrics, Harvard Medical School, Boston, MA, 02215, USA

## Background

Historically, social work education (SWE) has not included formal and indepth training to address substance use in either coursework or fieldwork. Health-care reform offers promise to integrate medical and behavioral health through employment of social work professionals. It is critical that screening, brief intervention, and referral to treatment (SBIRT) be incorporated as basic skills for all trainees, and other evidence-based practices be offered in SWE [1] to insure an adequate work force. This project describes design, implementation, and learner satisfaction of an adolescent SBIRT curriculum at Simmons School of Social Work (SSW) in partnership with Boston Children’s Hospital (BCH), through a Substance Abuse and Mental Health Services Administration (SAMHSA)-funded SBIRT training grant. We ask the question: “Are brief workshops and intensive practice courses feasible and acceptable methods of training social work students?”

## Methods

Participants were faculty members (n = 18), field advisers (n = 17), and master’s-level social work students (n = 34) at SSW. Training consisted of an SBIRT workshop offered to all faculty, field advisers, and students, which consisted of 3 core components: 1) impact of alcohol and marijuana on the developing adolescent brain; 2) adolescent SBIRT overview; and 3) brief motivational interventions. A social work practice course on the biopsychosocial dynamics of substance use assessment and treatment was also offered as an elective. The course incorporated the SBIRT workshop, observation at the BCH Adolescent Substance Abuse Program, and skill-based role play. We evaluated learner satisfaction in the workshop and practice course through the Government Performance and Results Act (GPRA) [2], administered immediately after training. For students in the practice course, we also administered the SSW course evaluation (25 items) at the end of the course.

## Results

Participants (N = 69) reported high rates of satisfaction and utility of training on the GPRA. More than 85 percent agreed/strongly agreed on 7/7 items immediately post-training, including that training was relevant to both substance abuse treatment and their career, enhanced their skills, and would benefit their clients. Students in the practice course reported high rates of satisfaction on 11 items across five social work core competencies [3] (*Engage*, *Assess*, *Intervene; Social Justice; Critical Thinking; Ethics and Professionalism*). In the SSW survey, neurobiology and motivational interviewing were frequently cited as “most helpful content to development as a professional social worker.” One student said, “*I finally feel like I know what I am doing thanks to the role plays we did in class.”*

## Conclusion

Brief workshops and intensive practice courses are feasible and acceptable methods of incorporating substance use training into SWE.
